# Supplementary material for: Sampling from Dirichlet process mixture models with unknown concentration parameter: mixing issues in large data implementations
Source: Stat Comput. 2014 May 3;25(5):1023–37. doi: 10.1007/s11222-014-9471-3 (PMC4550296; doi:10.1007/s11222-014-9471-3)
Supplement: Supplementary file 1 — Supplementary material 1 (pdf 82 KB) [file 11222_2014_9471_MOESM1_ESM.pdf]

Supplementary Material - Sampling from Dirichlet process mixture models with unknown concentration parameter: Mixing issues in large data implementations

David I. Hastie Silvia Liverani Sylvia Richardson

April 12, 2014

1 Simulated data

Here we provide additional details describing how we simulated the datasets that were used to illustrate our work.

1.1 Dataset 1 - used for paper Figure 1

This dataset is a simple simulation using aspects of a profile regression model, conditioning on 1000 observations being split into 5 clusters, each containing 200 observations. We assume a Bernoulli response model and 10 discrete covariates, each with 2 categories. No fixed effects or missing data points were included.

For each cluster c, the response and covariate data for the 200 observations were generated by sampling from the model using the the response parameter θ_c and the covariate model parameters Φ_c in Table 1 below. The values are fixed to ensure that the clusters were well separated within the parameter space.

	Cluster 1	Cluster 2	Cluster 3	Cluster 4	Cluster 5
θ_c	-2.19	-0.84	0	0.84	2.19
$\mathbb{P}(Y = 1)$	0.1	0.3	0.5	0.7	0.9
$\mathbb{P}(X_1 = 0)$	0.9	0.9	0.1	0.1	0.1
$\mathbb{P}(X_2 = 0)$	0.9	0.9	0.9	0.1	0.1
$\mathbb{P}(X_3 = 0)$	0.9	0.9	0.1	0.1	0.1
$\mathbb{P}(X_4 = 0)$	0.9	0.9	0.9	0.1	0.1
$\mathbb{P}(X_5 = 0)$	0.1	0.9	0.1	0.1	0.9
$\mathbb{P}(X_6 = 0)$	0.1	0.9	0.9	0.1	0.9
$\mathbb{P}(X_7 = 0)$	0.1	0.9	0.1	0.1	0.9
$\mathbb{P}(X_8 = 0)$	0.1	0.9	0.9	0.1	0.9
$\mathbb{P}(X_9 = 0)$	0.9	0.9	0.1	0.9	0.9
$\mathbb{P}(X_{10} = 0)$	0.1	0.1	0.9	0.1	0.1

Table 1: Response and covariate parameters used to generate simulated dataset 1.

2 Dataset 2 - used for paper Figure 9

The second dataset is generated more faithfully from a profile regression model, allowing the cluster allocations and cluster parameters to be generated according to their prior distributions. We simulate $n = 2,000$ observations. Each individual i has a discrete covariate vector X_i of $J = 10$ covariates (with each covariate sampled from one of $K_j = 5$ categories), a vector W_i of $L = 10$ fixed effects, and a Bernoulli outcome Y_i.

We use the following algorithm to generate the data.

- A
  - A.1 Set $C^\star = 1$, $C = 1$, $i = 0$, and $\psi_1 = 0$
  - A.2 If $i = n$ go to B
  - A.3 Set $i = i + 1$
  - A.4 Simulate $u \sim \text{Unif}(0, 1)$
  - A.5 If $u < \sum_{c<=C} \psi_c$ set $z_i = C$, $C = 1$ and go to A.2
  - A.6 If $C < C^\star$ set $C = C + 1$ and go to A.5
  - A.7 Simulate $\alpha_{C^\star} \sim \text{Gamma}(\text{shape} = 9, \text{scale} = 0.5)$
  - A.8 Simulate $v_{C^\star} \sim \text{Beta}(1, \alpha_{C^\star})$
  - A.9 Compute $\psi_{C^\star} = v_{C^\star} \prod_{l<C^\star}(1 - v_l)$
  - A.10 Set $C = C + 1$, $C^\star = C^\star + 1$ and go to A.5
- B For $c = 1, \ldots, C^\star$, we generate $\theta_c \sim t_7(0, 1)$ for the outcome and $\phi_{c,j} \sim \text{Dirichlet}(1, 1, 1, 1, 1)$ for $j = 1, \ldots, J$ for the covariates
- C For $l = 1, \ldots, L$ generate $\beta_l \sim t_7(0, 1)$ for the fixed effects coefficients.
- D For $i = 1, \ldots, n$ generate the fixed effect data W_i from a $\text{Normal}_L(0, I_L)$
- E For $i = 1, \ldots, n$ compute $\lambda_i = \theta_{z_i} + \beta W_i$.
- F For $i = 1, \ldots, n$ generate Y_i where $\mathbb{P}(Y_i = 1) = \text{logit}^{-1}(\lambda_i)$.
- G For $i = 1, \ldots, n$ generate $X_{i,j}$ according to the probabilities $\phi_{z_i,j}$, for each $j = 1, \ldots, J$.

3 Computation of marginal partition posterior

Here we provide further details regarding the computation of the marginal partition posterior, defined in Section 4.1 of the paper. To illustrate the computation we use the profile regression model used in the paper with a Bernoulli response and discrete categorical covariates.

Most generally, the marginal partition posterior is defined as $p(\boldsymbol{Z}|\boldsymbol{D})$.

For the case of profile regression, our data $\boldsymbol{D} = (\boldsymbol{Y}, \boldsymbol{X})$ is split into the response data $\boldsymbol{Y}$ and the covariate data $\boldsymbol{X}$. We must also condition on the fixed effect data $\boldsymbol{W}$. We can write

$$p(\boldsymbol{Z}|\boldsymbol{D}, \boldsymbol{W}) = p(\boldsymbol{Z}|\boldsymbol{Y}, \boldsymbol{X}, \boldsymbol{W}) \propto p(\boldsymbol{X}|\boldsymbol{Z})p(\boldsymbol{Y}|\boldsymbol{Z}, \boldsymbol{W})p(\boldsymbol{Z}).$$

The three factors on the right hand side are (from left to right) the likelihood of the covariate data, the likelihood of the response data and the prior for the partition. To proceed we look at each of these in turn.

3.1 Likelikood of covariate data

First we consider $p(\boldsymbol{X}|\boldsymbol{Z})$. Clearly computation of this factor depends entirely upon the covariate model. In the case considered in this paper the covariate model is a discrete categorical model, meaning we can write

$$p(\boldsymbol{X}|\boldsymbol{Z}) = \int p(\boldsymbol{X}|\Phi, \boldsymbol{Z})p(\Phi)\mathrm{d}\Phi.$$

This gives

$$p(\boldsymbol{X}|\boldsymbol{Z}) = \int \left[\prod_{i=1}^{n}\prod_{j=1}^{J}\phi_{z_i,j,x_{ij}}\right]\prod_{c=1}^{\infty}\prod_{j=1}^{J}\left[\frac{\Gamma(K_j a)}{\prod_{k=1}^{K_j}\Gamma(a)}\prod_{k=1}^{K_j}\phi_{c,j,k}^{a-1}\right]\mathrm{d}\Phi$$

where for $c = 1, 2, \ldots,$

$$p(\phi_{c,j}) \sim \text{Dirichlet}(\underbrace{a, \ldots, a}_{K_j \text{ times}})$$

and K_j is the number of categories for covariate j.

For empty clusters the integral is simply the integral of Φ_c over its prior. Since there is can only be a finite number of non-empty clusters, the infinite product can be written as a finite product meaning

$$p(\boldsymbol{X}|\boldsymbol{Z}) = \int \left[\prod_{i=1}^{n}\prod_{j=1}^{J}\phi_{z_i,j,x_{ij}}\right]\prod_{c:n_c>0}\prod_{j=1}^{J}\left[\frac{\Gamma(K_j a)}{\prod_{k=1}^{K_j}\Gamma(a)}\prod_{k=1}^{K_j}\phi_{c,j,k}^{a-1}\right]\mathrm{d}\Phi,$$

where n_c is the number of observations in cluster c.

This is the integral over a finite product of unnormalised Dirichlet distributions, giving

$$p(\boldsymbol{X}|\boldsymbol{Z}) = \prod_{c:n_c>0}\prod_{j=1}^{J}\left[\frac{\Gamma(K_j a)}{\prod_{k=1}^{K_j}\Gamma(a)}\frac{\prod_{k=1}^{K_j}\Gamma(a+n_{c,j,k})}{\Gamma(K_j a+n_c)}\right]$$

where $n_{c,j,k} = \sum_i 1_{\{z_i=c\}\cap\{x_{ij}=k\}}$.

3.2 Likelikood of response data

Next we consider the $p(\boldsymbol{Y}|\boldsymbol{Z},\boldsymbol{W})$. For the model considered in the paper, we assume a Bernoulli response, so that

$$\begin{aligned} p(\boldsymbol{Y}|\boldsymbol{Z},\boldsymbol{W}) &= \int p(\boldsymbol{Y}|\theta,\beta,\boldsymbol{Z},\boldsymbol{W})p(\theta)p(\beta)\mathrm{d}\theta\mathrm{d}\beta \\ &= \int \prod_{i=1}^{n} \left(\frac{e^{\theta_{z_i}+\beta^T\boldsymbol{W}}}{1+e^{\theta_{z_i}+\beta^T\boldsymbol{W}}}\right)^{Y_i} \left(\frac{1}{1+e^{\theta_{z_i}+\beta^T\boldsymbol{W}}}\right)^{1-Y_i} p(\theta)p(\beta)\mathrm{d}\theta\mathrm{d}\beta \end{aligned}$$

This is not a standard distribution so we use a multivariate Laplace approximation to approximate the integral. See Shun and McCullagh (1995) for a discussion of the magnitude and relevance of the error induced by the Laplace approximation. For a d-vector η, a function $h : \mathbb{R}^d \to \mathbb{R}$, and a large number M the multivariate Laplace approximation can be written as

$$\int e^{-Mh(\eta)}\mathrm{d}\eta \approx e^{-Mh(\hat{\eta})}|\Sigma|^{\frac{1}{2}}M^{\frac{d}{2}}(2\pi)^{\frac{1}{2}}$$

where $\hat{\eta}$ is the global minimum of h and Σ is the inverse Hessian of h evaluated at $\hat{\eta}$. Noting that we only need $p(\boldsymbol{Y}|\boldsymbol{Z},\boldsymbol{W})$ up to a constant, we can discard the final factor of the right hand side. Then, defining $M = n$ and $\eta = (\theta^\star, \beta)$, where $\theta^\star$ contains the elements of θ corresponding to non-empty clusters (the other θ's are just integrated over the prior) we obtain

$$p(\boldsymbol{Y}|\boldsymbol{Z},\boldsymbol{W}) \propto e^{-nh(\hat{\eta})}|\Sigma|^{1/2}n^{\frac{1}{2}(C^\star+L)} \qquad (1)$$

where $C^\star$ is the number of non-empty clusters and L is the number of fixed effects. The function h is given by

$$h(\eta) = -\frac{1}{n}\left(\sum_{i=1}^{n}\left[Y_i(\theta_{z_i}+\beta^T W_i) - \log(1+e^{\theta_{z_i}+\beta^T W_i})\right] + \log p(\theta^\star) + \log p(\beta)\right)$$

with

$$\log p(\theta^\star) = -\sum_{c:n_c>0}^{C} \frac{\nu+1}{2}\log\frac{\nu+\frac{\theta_c^2}{\sigma_\theta^2}}{\nu} + \text{constants}$$

and

$$\log p(\beta) = -\sum_{l=1}^{L} \frac{\nu+1}{2}\log\frac{\nu+\frac{\beta_l^2}{\sigma_\beta^2}}{\nu} + \text{constants},$$

where ν is the number of degrees of freedom of the t-distributions for θ_c and β_l.

To compute the exact value we thus proceed by

1. Finding $\hat{\eta}$, the value of η that minimises h

2. Evaluating h and the Hessian of h at $\hat{\eta}$
3. Plugging these into Equation (1)

3.3 Prior of partition

The final factor is the prior distribution of the partition $p(\boldsymbol{Z})$. This can be written as:

$$p(\boldsymbol{Z}) = \int p(\boldsymbol{Z}|\boldsymbol{V})p(\boldsymbol{V}|\alpha)p(\alpha)\mathrm{d}\boldsymbol{V}\mathrm{d}\alpha.$$

In order to simplify this calculation, we assume a fixed value of $\alpha = \alpha^\star$, which in our case was chosen as the posterior mean of our MCMC sample. Thus we can write

$$\begin{aligned} p(\boldsymbol{Z}) &= \int p(\boldsymbol{Z}|\boldsymbol{V})p(\boldsymbol{V}|\alpha^\star)\mathrm{d}\boldsymbol{V} \\ &= \int \prod_{i=1}^{n}\left[V_{z_i}\prod_{c<z_i}(1-V_c)\right]\prod_{c=1}^{\infty}\frac{1}{B(1,\alpha^\star)}(1-V_c)^{\alpha^\star-1}\mathrm{d}\boldsymbol{V}, \end{aligned}$$

where $B(a,b)$ denotes the Beta function.

Defining $C = \max_i z_i$, then

$$p(\boldsymbol{Z}) = \int \prod_{i=1}^{n}\left[V_{z_i}\prod_{c<z_i}(1-V_c)\right]\prod_{c=1}^{C}\frac{1}{B(1,\alpha^\star)}(1-V_c)^{\alpha^\star-1}\mathrm{d}V_1\mathrm{d}V_2\ldots\mathrm{d}V_C$$

since for the other empty clusters we are integrating V over the prior.

Following Lijoi et al. (2008), it follows that

$$p(\boldsymbol{Z}) = \frac{n!\Gamma(\alpha^\star)}{\Gamma(\alpha^\star+n)}\prod_{j=1}^{n}\frac{(\alpha^\star)^{a_j}}{j^{a_j}a_j!}$$

where $a_j = \#\{c : n_c = j\}$.

Additional References

Lijoi A, Pruenster I, Walker SG. (2008). Bayesian nonparametric estimators derived from conditional Gibbs structures. The Annals of Applied Probability, 18, 1519-1547

Shun, Zhenming, and P. McCullagh. "Laplace approximation of high dimensional integrals." Journal of the Royal Statistical Society. Series B (Methodological) (1995): 749-760.
